# Unilateral Graves’ Disease Developing During Treatment With Tirzepatide in a Patient With Diabetes: A Case Report

**DOI:** 10.1155/crie/9628050

**Published:** 2026-08-30

**Authors:** Yusuke Deto, Ryo Hasegawa, Shoya Ino, Satoru Shibata, Kouhei Ohno, Takahito Itoh, Tomoaki Matsumoto, Takayuki Miki

**Affiliations:** ^1^ Department of Cardiology and Diabetes, Oji General Hospital, Tomakomai, Japan

**Keywords:** adverse effect, case report, diabetes mellitus, tirzepatide, unilateral Graves’ disease

## Abstract

**Background:**

Tirzepatide is a dual agonist of the glucose‐dependent insulinotropic polypeptide (GIP) receptor and glucagon‐like peptide‐1 (GLP‐1) receptor, demonstrates significant efficacy in lowering blood glucose levels and reducing body weight. Consequently, its use has increased among patients with type 2 diabetes and obesity. Gastrointestinal symptoms are commonly recognized as adverse effects of tirzepatide, while other adverse effects, such as thyroid abnormalities, are rare. This report describes a rare case of unilateral Graves’ disease that developed during treatment with tirzepatide.

**Case Presentation:**

A 59‐year‐old woman with type 2 diabetes was treated with tirzepatide resulting in marked glycemic improvement and weight loss. During treatment, she developed fatigue and palpitations, initially attributed to the effects of the drug. Further evaluation revealed thyrotoxicosis with suppressed thyroid‐stimulating hormone (TSH), elevated free thyroid hormones, and positive autoantibodies. Imaging revealed increased vascularity and technetium‐99 m uptake confined to the right thyroid lobe, consistent with unilateral Graves’ disease. The patient’s clinical symptoms and thyroid function rapidly improved after initiating antithyroid medications.

**Conclusions:**

Although a causal relationship between tirzepatide and the development of Graves’ disease cannot be established, this case highlights the difficulty of recognizing hyperthyroidism during potent glucose‐lowering therapy. Thus, thyroid function testing should be considered in patients with type 2 diabetes who experience symptoms such as fatigue or palpitations during treatment with tirzepatide.

## 1. Introduction

Tirzepatide is a dual agonist of the glucose‐dependent insulinotropic polypeptide (GIP) receptor and glucagon‐like peptide‐1 (GLP‐1) receptor; this enhances insulin secretion, reduces glucagon levels, and promotes appetite suppression and weight loss [1, 2]. Compared to other antidiabetic medications, tirzepatide has significantly greater efficacy in lowering blood glucose levels and reducing body weight [1, 2]. Consequently, its use for treating patients with obese type 2 diabetes is rapidly increasing. Its adverse events commonly include gastrointestinal symptoms such as nausea, vomiting, constipation, and diarrhea. Cholelithiasis, cholecystitis, and pancreatitis have also been reported, albeit infrequently, whereas other specific adverse events such as thyroid abnormalities are rare [1–3]. Since substantial glucose lowering and weight loss can occur during treatment with tirzepatide, symptoms of hyperthyroidism such as mild fatigue or palpitations may be misattributed to the therapeutic effects of the drug, making the diagnosis difficult.

Graves’ disease is a common autoimmune thyroid disorder characterized by diffuse goiter, thyrotoxicosis, and ophthalmopathy, occasionally with infiltrative dermatopathy. Its symptoms, such as weight loss, palpitations, sweating, and hand tremors, are mainly related to excess thyroid hormone. This occurs due to homogeneous exposure of the thyroid gland to circulating thyroid‐stimulating hormone (TSH) receptor autoantibodies. Graves’ disease is typically diagnosed based on the clinical symptoms of thyrotoxicosis alongside the presence of autoantibodies, diffuse goiter, sonographic hypervascularity, and diffusely increased uptake on thyroid scintigraphy [4, 5]. Unilateral Graves’ disease is rare and may indicate an early stage of bilateral Graves’ disease, potentially remaining limited to mild symptoms of thyrotoxicosis [6, 7].

Although a causal relationship between tirzepatide and the development of Graves’ disease cannot be established, this case report describes a patient with type 2 diabetes who developed unilateral Graves’ disease during treatment with tirzepatide, initially attributing the clinical symptoms to the drug’s effects.

## 2. Case Presentation

**Figure 1:**
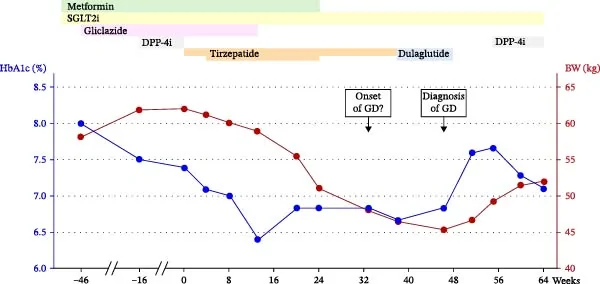
Clinical course of glycemic control, body weight, and medications for diabetes in the present case. Blue dots and line indicate HbA1c and red dots and line indicate body weight. DPP‐4i, dipeptidyl peptidase‐4 inhibitor; GD, Graves’ disease; SGLT2i, sodium‐glucose co‐transporter type 2 inhibitor.

**Figure 2:**
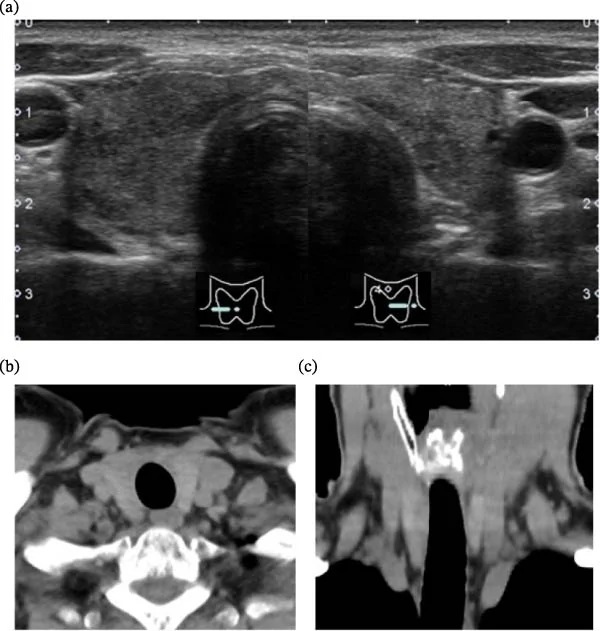
High‐resolution ultrasound of the thyroid (a), transverse view. The right lobe is slightly enlarged with a heterogenous echotexture, hypervascularity, and no nodules. The left lobe showed no enlargement, with no cysts or increased blood flow. Computed tomography scan of the thyroid, transverse (b) and coronal (c) views. The right lobe demonstrates mild enlargement.

**Figure 3:**
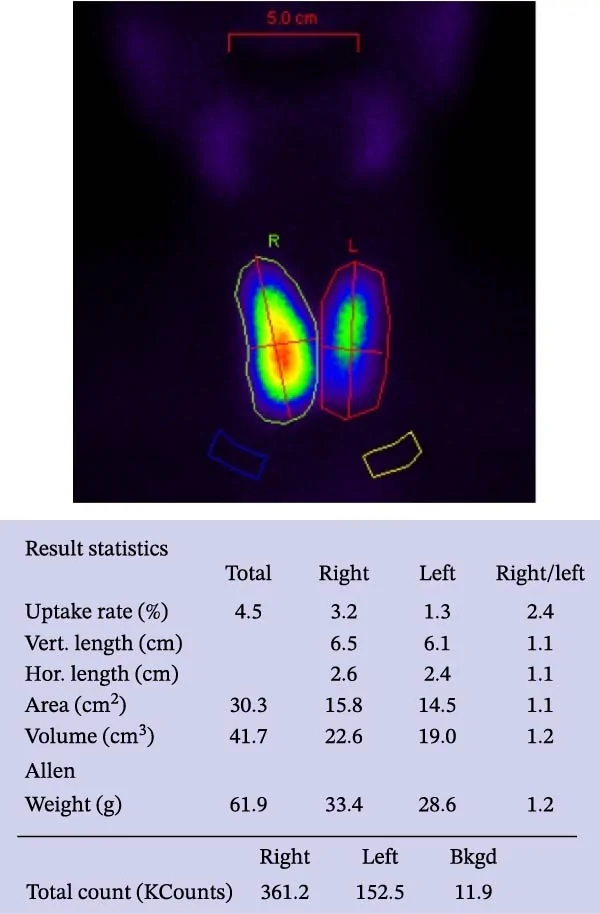
Technetium‐99 m thyroid scintigraphy demonstrating homogeneous uptake in the right lobe. The right and left lobes demonstrated an uptake of 3.2% and 1.3%, respectively.

A 59‐year‐old woman was referred to our hospital 2 years ago for the management of type 2 diabetes. At the initial visit, the patient had a height of 156.2 cm, weight of 57.8 kg, and body mass index of 23.7 kg/m^2^. Her medical history included left partial lung resection without postoperative chemotherapy for localized lung adenocarcinoma 3 years earlier. Her father has diabetes, but there is no family history of thyroid or autoimmune disease. She had been treated for diabetes at the referring institution with metformin and canagliflozin; however, glycemic control remained inadequate, with the HbA1c level above 8.0%. At our initial evaluation, laboratory tests revealed negative anti‐glutamic acid decarboxylase antibody, fasting plasma glucose level of 169 mg/dL, and C‐peptide level of 2.1 ng/mL. Thyroid function was normal; TSH: 0.74 µIU/mL (normal range 0.50–5.00 µIU/mL), and free T4: 1.42 ng/dL (normal range 0.90–1.70 ng/dL). Blood pressure and lipid levels were well‐controlled with olmesartan, rosuvastatin, and ezetimibe. Gliclazide and a dipeptidyl peptidase‐4 (DPP‐4) inhibitor were added sequentially, resulting in a modest decrease in HbA1c to 7.4% and weight gain to 61.9 kg (Figure 1). Subsequently, the DPP‐4 inhibitor was discontinued, and tirzepatide was initiated at 2.5 mg/week, which was then increased to 5.0 mg/week after 4 weeks. Gliclazide was discontinued after HbA1c improved to 6.4%, which remained below 7.0% thereafter. The body weight decreased to 51.1 kg over 6 months. Because of marked appetite loss, metformin was discontinued and tirzepatide dose was reduced to 2.5 mg/week. Despite these adjustments, the patient’s weight further declined to 46.1 kg after 4 months, accompanied by fatigue and palpitations. Tirzepatide was switched to dulaglutide. Since blood pressure was also low, olmesartan was discontinued, and bisoprolol was initiated at 1.25 mg/day. After an additional 2 months, the patient’s HbA1c remained stable at 6.8%, but her body weight decreased to 45.3 kg. Electrocardiogram revealed sinus rhythm with a heart rate of 88 beats/min, while echocardiography demonstrated normal cardiac function with a left ventricular ejection fraction of 69%. Laboratory testing revealed thyrotoxicosis, with TSH < 0.01 µIU/mL (normal range: 0.50–5.00 µIU/mL), free T4 of 3.55 ng/dL (normal range: 0.90–1.70 ng/dL), and free T3 of 11.6 pg/mL (normal range: 2.30–4.00 pg/mL). The patient also tested positive for TSH receptor antibody (2.9 IU/L, normal range <2.0 IU/L), thyroid‐stimulating antibody (352%, normal range <110%) and antithyroid peroxidase antibody (269 IU/mL, normal range <5.6 IU/mL). High‐resolution ultrasound and computed tomography showed slight enlargement and increased vascularity of the thyroid right lobe (Figure 2a–c), but the gland was not palpable on physical examination. Technetium‐99 m thyroid scintigraphy demonstrated a homogeneously increased uptake in the right lobe, with no increased uptake in the left lobe (Figure 3). The total uptake rate was 4.5% (normal range: 0.5%–4.0%). Based on these findings, the patient was diagnosed with unilateral Graves’ disease of the right lobe. Treatment with thiamazole was initiated, and bisoprolol was increased to 2.5 mg/day. Four months later, the patient’s thyroid function improved; TSH: 1.84 µIU/mL (normal range 0.50–5.00 µIU/mL), free T4: 0.87 ng/dL (normal range 0.90–1.70 ng/dL) and free T3: 2.43 pg/mL (normal range 2.30–4.00 pg/mL). The body weight recovered to 52.1 kg and symptoms such as fatigue and palpitations resolved.

## 3. Discussion

The most frequently reported adverse events with tirzepatide are gastrointestinal symptoms such as nausea, vomiting, constipation, and diarrhea [1–3, 8]. Cholelithiasis, cholecystitis, and pancreatitis are less common adverse effects that warrant attention as well. Thyroid abnormalities are rare and not widely known, although these are listed in the drug information with provided warnings. Preclinical studies in rodents revealed that GLP‐1 receptor agonists and tirzepatide can promote C‐cell proliferation and increase the risk of thyroid cancer, including medullary thyroid carcinoma [8, 9]. However, meta‐analyses have yielded conflicting results regarding whether GLP‐1 agonists and tirzepatide increase the risk of thyroid abnormalities (e.g., thyroid cancer) in humans [10–12]. Notably, medullary thyroid carcinoma expresses higher levels of GLP‐1 and GIP receptors compared to normal thyroid tissue [8], thus warranting caution in patients with a family history of medullary thyroid carcinoma or multiple endocrine neoplasia type 2.

Manueli Loas et al. [13] conducted a retrospective analysis of 527 patients treated with tirzepatide for 12 months to evaluate the incidence of new‐onset or worsening thyroid disease. Thyroid disorders were identified in 28 patients (5.3%), with thyroid nodules/goiter and drug‐induced thyroiditis being the most frequently reported conditions. Multivariable analysis demonstrated that pre‐existing thyroid disease and end‐stage renal disease were significant risk factors for the development or worsening of thyroid disorders, whereas no association was found between the tirzepatide dose and thyroid disease risk. To date, only three case reports have described thyroid dysfunction associated with tirzepatide [14–16]. One reported painless thyroiditis with transient thyrotoxicosis that resolved after the discontinuation of tirzepatide [14]. The others described worsening thyrotoxicosis in a patient receiving stable levothyroxine therapy following rapid weight loss [15, 16]. Given that weight loss itself can alter thyroid hormone levels and reduce thyroid hormone requirements [17, 18], it remains uncertain whether the observed thyrotoxicosis was directly attributable to tirzepatide or was secondary to weight loss induced by the drug. In addition, although one case of Graves’ disease was identified in the retrospective study by Manueli Loas et al. [13], no detailed clinical information was provided. In the present case, the patient developed Graves’ disease accompanied by positive thyroid autoantibodies. Therefore, thyrotoxicosis cannot be explained solely by alterations in thyroid hormone requirements resulting from weight loss. Nevertheless, a causal relationship between tirzepatide and the development of Graves’ disease cannot be established based on the currently available evidence.

Overall, evidence linking tirzepatide to thyroid dysfunction remains limited, and existing data do not adequately distinguish weight loss–mediated effects from direct drug‐specific effects. Several clinical manifestations observed in the present case, including weight loss, fatigue, and palpitations, may be attributable either to the expected effects of tirzepatide therapy or to thyrotoxicosis, potentially delaying the recognition of the underlying thyroid dysfunction. Clinicians should therefore remain vigilant for thyroid dysfunction, particularly in patients with pre‐existing thyroid disease or end‐stage renal disease. In addition to evaluating thyroid function in patients who develop symptoms suggestive of thyrotoxicosis, periodic monitoring of thyroid function may also be considered in patients with these risk factors [13]. When marked or unexpected weight loss occurs, particularly in patients receiving relatively low doses of tirzepatide, clinicians should broaden the differential diagnosis rather than attributing the finding solely to the medication. Conditions, such as hyperthyroidism, gastrointestinal disorders, and other systemic illnesses, should be evaluated based on the clinical context. In the present case, a comprehensive evaluation was performed to exclude other potential causes of substantial weight loss before Graves’ disease was diagnosed. Ultimately, careful history taking, thorough physical examination, and thoughtful consideration of the differential diagnosis remain essential to avoid overlooking an alternative or coexisting disease.

Graves’ disease is caused by circulating autoantibodies that bind to TSH receptors, resulting in thyroid hyperfunction and tissue proliferation [4, 5]. This typically results in diffuse bilateral goiter, whereas unilateral Graves’ disease confined to a single lobe is rare, with only a few reported cases [6, 7, 19–24]. Unilateral Graves’ disease has been described across a wide age range, but most patients are aged 30–50 years. This occurs more frequently in women, although cases in men have also been reported. The right lobe appears is more commonly affected as well [6, 7, 19–24]. The cause of unilateral Graves’ disease remains unclear, but several possibilities have been suggested. First, unilateral Graves’ disease may represent an early phase of bilateral Graves’ disease. This is suggested in two cases reported by Sakata et al. [6], wherein patients underwent right hemithyroidectomy but developed thyrotoxicosis with enlargement of the remaining left lobe after 27 and 8 months, respectively. Second, structural and functional asymmetry between the thyroid lobes may result in unilateral disease. Notably, the right lobe is generally larger than the left in healthy individuals [21], while congenital absence or hypoplasia occurs more frequently in the left lobe [25]. Thus, the right lobe is more dominant in terms of vascularity and development. In addition, the separate lymphatic drainage of each lobe may result in localized autoimmune responses [19, 20, 26]. Lastly, differences in sodium iodide symporter gene expression and function between the two lobes can contribute to impaired radioactive iodine uptake in the thyroid gland. Iodine reaches the gland for thyroid hormone biosynthesis through the sodium iodide symport, making it crucial in thyroid pathophysiology [27].

Unilateral Graves’ disease should follow the same treatment principles as those for conventional Graves’ disease. Thyroidectomy is generally not recommended, since there are reports of recurrence in the remaining lobe after surgical intervention [6]. Most reported cases, included the present case, respond well to antithyroid drugs [7, 19–21, 23, 24]. Nevertheless, long‐term follow‐up is needed to determine whether remission and antibody negativity can be achieved in this case.

## 4. Conclusion

This report describes a patient with type 2 diabetes who developed unilateral Graves’ disease during treatment with tirzepatide. Weight loss, gastrointestinal symptoms, and other accompanying symptoms, such as fatigue and palpitations, are often attributed to the pharmacological effects of tirzepatide. However, this case highlights the importance of remaining vigilant for thyroid‐related adverse events during tirzepatide treatment, even in patients with normal baseline thyroid function and a low risk of thyroid malignancy.

## Author Contributions

**Yusuke Deto**: manuscript writing, literature review. **Ryo Hasegawa, Shoya Ino, Satoru Shibata, Kouhei Ohno, Takahito Itoh, and Tomoaki Matsumoto:** data interpretation, literature review. **Takayuki Miki**: clinical care of the patient, manuscript writing and editing.

## Funding

No funding was received for this study.

## Disclosure

All authors have read and approved the final version of the manuscript. Takayuki Miki had full access to all of the data in this study and takes complete responsibility for the integrity of the data and the accuracy of the data analysis.

## Ethics Statement

This article does not contain any studies with human or animal subjects performed by any of the authors. This case report was approved by the Clinical Investigation Ethics Committee of Oji General Hospital (OGH2024‐48).

## Consent

Written informed consent was obtained from the patient.

## Conflicts of Interest

Takayuki Miki received honoraria for educational lectures from AstraZeneca K.K., Eli Lilly Japan K.K., Nippon Boehringer Ingelheim Co., Novartis Pharma K.K., Novo Nordisk Pharma Ltd., Otsuka Pharmaceutical Co., Ltd., and Tanabe Pharma Corporation, Ltd. The other authors declare no conflicts of interest.

## Data Availability

Data sharing is not applicable to this article, as no datasets were generated or analyzed during the current study.

## References

[1] Anderson S. L. and Marrs J. C. , Tirzepatide for Type 2 Diabetes, Drugs in Context. (2023) 12, 1–8, 10.7573/dic.2023-6-1. PMC10470858 37664792

[2] Vázquez L. A. , Tofé-Povedano S. , and Bellido-Guerrero D. , et al.Use of Tirzepatide in Adults With Type 2 Diabetes Mellitus: Scientific Evidence and Practical Aspects, Diabetes Therapy. (2024) 15, no. 7, 1501–1512, 10.1007/s13300-024-01587-6. 38722495 PMC11211290

[3] Caruso I. , Di Gioia L. , and Di Molfetta S. , et al.The Real-World Safety Profile of Tirzepatide: Pharmacovigilance Analysis of the FDA Adverse Event Reporting System (FAERS) Database, Journal of Endocrinological Investigation. (2024) 47, no. 11, 2671–2678, 10.1007/s40618-024-02441-z. 39141075 PMC11473560

[4] Singh S. K. , Singh R. , Pandey A. K. , Singh V. , Tiwari A. , and Rai P. K. , Recent Advances in Management of Graves’ Disease: An Update, Postgraduate Medical Journal. (2026) 102, no. 1206, 302–310, 10.1093/postmj/qgaf197. 41259222

[5] Tagami T. , Katai M. , and Kinuya S. , et al.Guidelines for the Diagnosis of Graves’ Disease, Hypothyroidism, Painless Thyroiditis, Chronic Thyroiditis (Hashimoto’s Thyroiditis), and Subacute Thyroiditis (Acute Phase), Thyroid Science. (2025) 2, no. 4, 10.1016/j.thscie.2025.100032, 100032.

[6] Sakata S. , Fuwa Y. , and Goto S. , et al.Two Cases of Graves’ Disease With Presentation of Unilateral Diffuse Uptake of Radioisotopes, Journal of Endocrinological Investigation. (1993) 16, no. 11, 903–907, 10.1007/BF03348954. 8144868

[7] Alaybaa K. and Alhuzaim O. , Unilateral Graves’ Disease in a Bilobar Thyroid Gland: A Very Unusual cause of Hyperthyroidism, JCEM Case Reports. (2023) 1, no. 3, 1–3, 10.1210/jcemcr/luad048. PMC10580439 37908571

[8] Fahim S. A. , Attia Y. M. , Messiha A. , Nabawy A. Y. , Refaat F. , and El-Maadawy W. H. , Comparative Safety and Side Effects of Semaglutide and Tirzepatide: Implications for Clinical Decision-Making in Obesity Management, Biomedicine & Pharmacotherapy. (2025) 193, 10.1016/j.biopha.2025.118731, 118731. 41177120

[9] van den Brink W. , Emerenciana A. , Bellanti F. , Della Pasqua O. , and van der Laan J. W. , Prediction of Thyroid C-Cell Carcinogenicity After Chronic Administration of GLP1-R Agonists in Rodents, Toxicology and Applied Pharmacology. (2017) 320, 51–59, 10.1016/j.taap.2017.02.010. 28213092

[10] Bezin J. , Gouverneur A. , and Pénichon M. , et al.GLP-1 Receptor Agonists and the Risk of Thyroid Cancer, Diabetes Care. (2023) 46, no. 2, 384–390, 10.2337/dc22-1148. 36356111

[11] Hu W. , Song R. , and Cheng R. , et al.Use of GLP-1 Receptor Agonists and Occurrence of Thyroid Disorders: A Meta-Analysis of Randomized Controlled Trials, Frontiers in Endocrinology. (2022) 13, 10.3389/fendo.2022.927859, 927859. 35898463 PMC9309474

[12] Kamrul-Hasan A. B. M. , Alam M. S. , Dutta D. , Sasikanth T. , Aalpona F. T. Z. , and Nagendra L. , Tirzepatide and Cancer Risk in Individuals With and Without Diabetes: A Systematic Review and Meta-Analysis, Endocrinology and Metabolism. (2025) 40, no. 1, 112–124, 10.3803/EnM.2024.2164. 39814031 PMC11898313

[13] Manueli Laos E. G. , Serafica B. , and Fontaine-Nicola A. , et al.Impact of Tirzepatide Therapy on Thyroid Disease: Understanding Risks and Emerging Insights, Clinical Obesity. (2026) 16, no. 3, 10.1111/cob.70084. 42145153

[14] Humaida S. , Manzalji K. , Seyam N. , and Al-Masalmani L. , Dual Glucagon-Like Peptide-1 (GLP-1) and Glucose-Dependent Insulinotropic Polypeptide (GIP) Receptor Agonist-Associated Thyroiditis: A Case Report of Thyroid Dysfunction Following Tirzepatide use, Cureus. (2025) 17, 10.7759/cureus.85123. PMC12127604 40458348

[15] Karakus K. E. , Shah V. N. , and Akturk H. K. , Tirzepatide-Induced Rapid Weight Loss–Related Thyrotoxicosis, JAMA Internal Medicine. (2024) 184, no. 10, 1246–1247, 10.1001/jamainternmed.2024.2919. 39102259

[16] Adams E. W. , Somers A. , Garrido-Cortes E. , and Wrights B. , Thyroid Dysfunction Following Tirzepatide use in a Post-Thyroidectomy Patient on Stable Levothyroxine Therapy: A Case Study, Cureus. (2026) 18, 10.7759/cureus.106680. PMC13155940 42109981

[17] Nayak S. S. , Hashemi S. M. , and Patel M. , et al.The Impact of Weight Loss Interventions on Thyroid Function: A Systematic Review and Meta-Analysis, Annals of Medicine & Surgery. (2025) 87, no. 7, 4484–4497, 10.1097/MS9.0000000000003428. 40852024 PMC12369776

[18] Santini F. , Pinchera A. , and Marsili A. , et al.Lean Body Mass Is a Major Determinant of Levothyroxine Dosage in the Treatment of Thyroid Diseases, The Journal of Clinical Endocrinology & Metabolism. (2005) 90, no. 1, 124–127, 10.1210/jc.2004-1306. 15483074

[19] Dimai H. P. , Ramschak-Schwarzer S. , and Lax S. , et al.Hyperthyroidism of Graves’ Disease: Evidence for Only Unilateral Involvement of the Thyroid Gland in a 31-Year-Old Female Patient, Journal of Endocrinological Investigation. (1999) 22, no. 3, 215–219, 10.1007/BF03343545. 10219891

[20] Gratz S. , Barth P. , Arnold R. , and Behr T. M. , Graves’ Disease With Unilateral Radioisotope Uptake, Nuklearmedizin. (2004) 43, no. 5, N66–N68. 15536692

[21] Bolognesi M. and Rossi R. , Case History: Unilateral Graves’ Disease, Thyroid. (2006) 16, no. 5, 493–495, 10.1089/thy.2006.16.493. 16756472

[22] Al Juhanni N. , Wagieh S. , and Al Ghamdi H. , Graves’ Disease Affecting One Thyroid Lobe (Unilateral Graves’ Disease): Case Report, Egyptian Journal of Radiology and Nuclear Medicine. (2010) 2, 62–65.

[23] Pande A. , Aggarwal A. , and Wadhwa R. , Unilateral Grave’s Disease: A Rare Variant, Journal of Clinical and Diagnostic Research. (2019) 13, OD06–OD08, 10.7860/JCDR/2019/42313.13147.

[24] Bshait M. S. B. , Graves’ Disease With Only Unilateral Involvement; a Case Report, International Journal of Surgery Case Reports. (2024) 114, no. C, 10.1016/j.ijscr.2023.109138, 109138. 38086135 PMC10758942

[25] Maiorana R. , Carta A. , and Floriddia G. , et al.Thyroid Hemiagenesis: Prevalence in Normal Children and Effect on Thyroid Function, The Journal of Clinical Endocrinology & Metabolism. (2003) 88, no. 4, 1534–1536, 10.1210/jc.2002-021574. 12679435

[26] Dayan C. M. and Daniels G. H. , Chronic Autoimmune Thyroiditis, New England Journal of Medicine. (1996) 335, no. 2, 99–107, 10.1056/NEJM199607113350206. 8649497

[27] De la Vieja A. , Dohan O. , Levy O. , and Carrasco N. , Molecular Analysis of the Sodium/Iodide Symporter: Impact on Thyroid and Extrathyroid Pathophysiology, Physiological Reviews. (2000) 80, no. 3, 1083–1105, 10.1152/physrev.2000.80.3.1083. 10893432

